# Ubiquinone (Coenzyme Q-10) Supplementation Influences Exercise-Induced Changes in Serum 25(OH)D_3_ and the Methyl-Arginine Metabolites: A Double-Blind Randomized Controlled Trial

**DOI:** 10.3390/antiox13070760

**Published:** 2024-06-23

**Authors:** Jan Mieszkowski, Andrzej Kochanowicz, Paulina Brzezińska, Magdalena Kochanowicz, Katarzyna Żołądkiewicz, Błażej Stankiewicz, Bartłomiej Niespodziński, Joanna Reczkowicz, Konrad Kowalski, Jędrzej Antosiewicz

**Affiliations:** 1 Department of Gymnastics and Dance, Gdańsk University of Physical Education and Sport, 80-336 Gdańsk, Polandkatarzyna.zolakiewicz@awf.gda.pl (K.Ż.); 2 Faculty of Physical Education and Sport, Charles University, 162 52 Prague, Czech Republic; 3 Department of Physical Therapy, Medical University of Gdańsk, 80-211 Gdańsk, Poland; magdalena.kochanowicz@gumed.edu.pl; 4 Department of Biomedical Basis of Physical Education, Institute of Physical Education, Kazimierz Wielki University, 85-064 Bydgoszcz, Poland; blasta@ukw.edu.pl; 5 Department of Biological Foundations of Physical Education, Faculty of Health Sciences and Physical Education, Kazimierz Wielki University, 85-064 Bydgoszcz, Poland; bartlomiej.niespodzinski@ukw.edu.pl; 6 Department of Bioenergetics and Physiology of Exercise, Medical University of Gdańsk, 80-211 Gdańsk, Poland; joanna.reczkowicz@gumed.edu.pl (J.R.); konrad.kowalski@masdiag.pl (K.K.)

**Keywords:** endurance exercise, 3-*epi*-25(OH)D_3_, 24,25(OH)_2_D_3_, 25(OH)D_3_, coenzyme Q_10_, ubiquinone

## Abstract

Researchers have studied the effects of exercise on serum methyl-arginine and vitamin D metabolites; however, the effects of exercise combined with antioxidants are not well documented. Since oxidative stress affects the metabolism of vitamin D and methyl-arginine, we hypothesised that the antioxidant coenzyme Q_10_ (CoQ_10_) might modulate exercise-induced changes. A group of twenty-eight healthy men participated in this study and were divided into two groups: an experimental group and a control group. The exercise test was performed until exhaustion, with gradually increasing intensity, before and after the 21-day CoQ_10_ supplementation. Blood samples were collected before, immediately after, and 3 and 24 h after exercise. CoQ_10_, vitamin D metabolites, asymmetric dimethylarginine (ADMA), symmetric dimethylarginine, methylarginine, dimethylamine, arginine, citrulline, and ornithine were analysed in serum samples. CoQ_10_ supplementation caused a 2.76-fold increase in the concentration of serum CoQ_10_. Conversely, the 25(OH)D_3_ concentration increased after exercise only in the placebo group. ADMA increased after exercise before supplementation, but a decrease was observed in the CoQ_10_ supplementation group 24 h after exercise. In conclusion, our data indicate that CoQ_10_ supplementation modifies the effects of exercise on vitamin D and methyl-arginine metabolism, suggesting its beneficial effects. These findings contribute to the understanding of how antioxidants like CoQ_10_ can modulate biochemical responses to exercise, potentially offering new insights for enhancing athletic performance and recovery.

## 1. Introduction

Coenzyme Q_10_ (CoQ_10_) is an important compound for cell energetic metabolism as part of the respiratory chain, and it has strong antioxidant properties [1]. CoQ_10_ is commonly used to prevent and treat diseases such as myocardial infarction, congestive heart failure, acute pancreatitis, and many other diseases [2,3]. Moreover, CoQ_10_ can prevent oxidative stress, including that induced by exhaustive exercise [3]. Conversely, vitamin D, although not considered an antioxidant, can promote the enzymatic antioxidant potential of cells [4].

It is well known that exercise increases free radical formation and markers of oxidative stress [5]. Increases in lipid and protein peroxidation products and other oxidative changes have been reported after exercise [6]. An increase in reactive oxygen species (ROS) formation can also affect some metabolic changes. For example, ROS can inactivate dimethylarginine dimethylaminohydrolase (DDAH)—an enzyme that is important for eliminating asymmetric dimethylarginine (ADMA) and methylarginine (MMA) [7], both of which compete with arginine for the active centre of nitric oxide synthase (NOS), thus blocking NO synthesis. Reduced NO formation can affect many processes, such as blood flow, glucose transport, and mitochondrial respiration, among others [8]. ROS can also influence vitamin D metabolism [9]. Low glucose-6-phosphate dehydrogenase activity leads to decreased formation of NADPH and a drop in intracellular glutathione (GSH), which negatively influences vitamin D activation and receptor-mediated action [10,11]. A single bout of exercise has been shown to increase serum level of 25(OH)D_3_. However, it is unknown if this is a result, e.g., augmented lipolysis, as part of vitamin D is stored in adipose tissue, or a stress response manifested by increased vitamin D hydroxylation. Both vitamin D hydroxylases and CoQ_10_ are located mainly in mitochondria; thus, it is possible that they can influence each other. While CoQ_10_ is recognised for its role in reducing oxidative damage and improving cellular energy production, the specific interactions with vitamin D and pathways through which it exerts its protective effects remain to be fully elucidated. As mentioned above, CoQ_10_ has been reported to reduce exercise-induced oxidative stress, but there are no reports demonstrating that it can inhibit exercise lipolysis.

Thus, in this study, we postulated that CoQ_10_ will abolish exercise-induced changes in 25(OH)D_3_. Moreover, we hypothesised that an exercise-induced increase in ADMA, as observed in previous studies [12], would be abrogated by CoQ_10_ supplementation. Therefore, the study aimed to evaluate the effects of CoQ_10_ supplementation on exercise-induced changes in serum 25(OH)D_3_ and methyl-arginine metabolites.

## 2. Materials and Methods

### 2.1. Ethics

The Bioethics Committee for Clinical Research at the Regional Medical Chamber in Gdansk approved this research (Consent No. KB-23/22), which was conducted according to the principles of the Declaration of Helsinki. Prior to the study, the participants gave written informed consent to participate, and they had the option of withdrawing at any time for any reason. The study was registered as a clinical trial NCT05412888.

### 2.2. Experimental Overview

**Figure 1:**
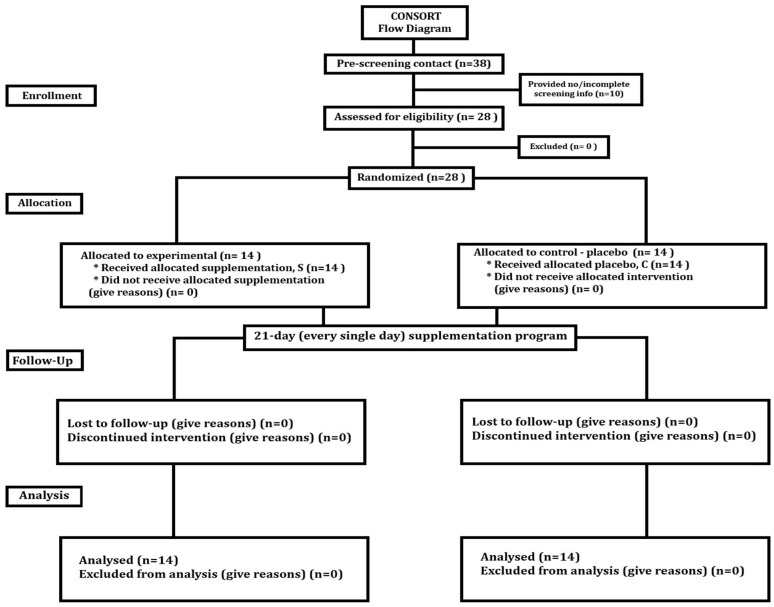
Participants flow diagram.

The study was designed as a double-blind, randomised, controlled trial with parallel groups (Figure 1). The supplementation protocol involved a 21-day (7 times per week) supplementation program with 300 mg of CoQ_10_.

During the initial study visits, the participants’ ages, body compositions, and heights were recorded. All participants were examined by a professional physician and found to be healthy. Before and after 21 days of supplementation, the participants completed the Bruce Treadmill Aerobic Test (BTT). Furthermore, to evaluate how CoQ_10_ supplementation modifies the effects of exercise on vitamin D and methyl-arginine metabolism, venous blood samples were collected at selected timepoints before and after the supplementation period for serum analysis. All laboratory analyses were performed at the Gdansk University of Physical Education (Gdansk, Poland).

### 2.3. Participants

Twenty-eight physically active, healthy men participated in the study. They all regularly participated in recreational sports, such as running, swimming, judo, and team sports (on average, 2–3 times per week for 45 min each time). The participants had normal health status during the preceding three months, defined as no bone or muscle tissue injuries, no history of abnormalities of the cardiovascular system or autonomic nervous system, and no history of mental disorders or other diseases that might directly affect the study results.

The participants took no drugs during the study or in the three months preceding it and were provided with a list of foods rich in CoQ_10_ that could not be eaten during the experiment. Prior to the start of the study, a medical practitioner examined all participants. They were informed about the study procedures before enrolment but were unaware of the study aims or the supplementation schedule. The runners were randomly assigned to two groups: an experimental (supplementation, S; n = 14, age 20.11 ± 1.12 years) and a control (placebo, C; n = 14, age 20.32 ± 1.57 years).

### 2.4. Measurement of Aerobic Components of Fitness: Bruce Treadmill Test

The Bruce protocol was performed on an electric treadmill (H/P/Cosmos, München, Germany). After standardising the warm-up (5 min with 60% HR max), each participant ran with increasing loads induced by velocity and treadmill inclination. During the testing, participants underwent specific test stages from 1 to 10 with increasing speed and velocity, from a 10% incline at 2.7 km/h (level 1) to a 28% incline at 12.07 km/h (level 10). Time was constant for each stage, with velocity and inclination alterations at every three minutes. The test was stopped when the subject could no longer continue owing to fatigue or other conditions. The interruption criterion was associated with fatigue, difficulties in breathing, muscular tiredness, chest pain or any factor limiting the effort. The whole treadmill speed and incline are presented in the Table 1. The test was stopped when the subject could no longer continue owing to fatigue or other conditions.

### 2.5. Supplementation

Both groups completed a 21-day (every single day) supplementation program. The experimental population received 300 mg of CoQ_10_.

The control group received the same number of identical-looking, rice-flavoured gelatine capsules. The CoQ_10_ and placebo supplements were both produced by the CoQ_10_-cyclodextrin complex as HydroQSorb^®^ (Tishcon Corp, New York, NY, USA), and they were placed in identical plastic boxes marked by codes indicating the type of preparation and recommended dose. Participants did not know what kind of supplement they received.

### 2.6. Sample Collection and Measurements of CoQ_10_, Vitamin D, ADMA, Symmetric Dimethylarginine (SDMA), MMA, Dimethylamine (DMA), Arginine (Arg), Citrulline (Cit), and Ornithine (Orn)

Blood samples were collected (in a sitting position) before and after 21 days of CoQ_10_ supplementation. Whole blood samples were collected at four timepoints (immediately before, after, 3 h and 24 h after a BTT) in 5 mL BD Vacutainer Clot Activator Tubes (Becton Dickinson and Company, Franklin Lakes, NJ, USA). The serum was separated by centrifugation at 4000× *g* for 10 min and aliquoted into 500 μL portions. The samples were frozen and stored (for no longer than 6 months) at −80 °C until further analysis.

Sample preparation was based on serum protein precipitation and derivatisation: 4-(4′-Dimethylaminophenyl)-1,2,4-triazoline-3,5-dione, used as the derivatisation agent, was synthesised at the Masdiag Laboratory (Warsaw, Poland). Quantitative analysis of vitamin D metabolites was performed using liquid chromatography coupled with tandem mass spectrometry (LC-MS/MS) (QTRAP^®^4500 (Sciex, Framingham, MA, USA) coupled with an ExionLC HPLC system) according to Rola et al. [13], with minor changes. Methyl-arginine metabolites and CoQ_10_ were also analysed using LC-MS/MS.

Serum samples were analysed in positive ion mode using electrospray ionisation. The raw data were collected using LabSolutions LCGC, which was also used to process and quantify the collected data. Mobile phases were prepared with acetonitrile (Honeywell, Sigma-Aldrich, Gillingham, Dorset, UK), water (POCh S.A., Gliwice, Poland), and formic acid (Merck KGaA, Darmstadt, Germany). All solvents were LC-MS-grade.

The following vitamin D metabolites were analysed: 25(OH)D_3_, 25(OH)D_2_, 24,25(OH)_2_D_3_, 3-*epi*-25(OH)D_3_, 24,25(OH)_2_D_3_:25(OH)D_3_ ratio, and 3-*epi*-25(OH)D_3_:25(OH)D_3_ ratio. All the concentrations of vitamin D metabolites are expressed as ng/mL, which can be transformed to nmol/L by multiplying by 2.5, e.g., 30 ng/mL 25(OH)D_3_ equals 75 nmol/L. The following methyl-arginine metabolites were also analysed: ADMA, Arg, Cit, DMA, MMA, Orn, Arg:ADMA ratio and SDMA.

The concentration of CoQ_10_ was determined using reverse-phased high-performance liquid chromatography coupled with tandem mass spectrometry. Quantitative analysis involved calculating the ratio of the amount of an internal standard of known concentration to the amount of analyte read from the standard curve. A second MRM pass (qualitative analysis) confirmed the compound’s identity.

### 2.7. Statistical Analysis

Descriptive statistics included the mean ± standard deviation (SD) for all measured variables. The data were analysed using a set of two two-factor mixed-design analyses of variance (ANOVA). In the first analysis, the within-subject factor (repeated measures) represented the supplementation effect on the performance variables. In the second analysis, the within-subject factor (repeated measures) represented the effort effect of BTT (at baseline, immediately after BTT, and 3 h and 24 h after BTT), and the between-subject factor represented the group effect (supplementation or control group). The significance threshold was set at *p* < 0.05. The required total sample sizes of 12 (ANOVA: 2 × 4) and 16 participants (ANOVA: 2 × 2) for the groups were estimated using G*Power v. 3.1.9.4. software (Franz Faul et al., Universität Kiel, Kiel, Germany) for a large effect size, and a power of 0.80. Statistical analysis was performed using Statistica 13 software (TIBCO Software Inc., Palo Alto, CA, USA).

## 3. Results

### 3.1. Anthropometric Characteristics and Performance

The performance and physiological characteristics of the groups before and after 21 days of supplementation with CoQ_10_ are shown in Table 2. The examined morphological and physiological parameters showed no significant changes after 21 days of CoQ_10_ supplementation.

### 3.2. Resting Values before and after the 21-Day Supplementation Period

Resting concentrations of CoQ_10_, vitamin D_3_ metabolites, and amino acid metabolites before and after 21 days of supplementation with CoQ_10_ are shown in Table 3.

A two-way ANOVA with repeated-measure analysis showed a significant increase over 2.7 times baseline CoQ_10_ after 21 days of CoQ_10_ supplementation. In the supplementation group, significant increases in the baseline concentrations of 25(OH)D_3_ (17.3%, *p* < 0.01) 3-*epi*-25(OH)D_3_ (53.9%, *p* < 0.01), 3-*epi*-25(OH)D_3_:25(OH)D_3_ ratio (32.0%, *p* < 0.01), Arg (12.0%, *p* < 0.05), MMA (24.7%, *p* < 0.01), and SDMA (16.0 %, *p* < 0.01) were also observed.

In addition, the resting concentrations of CoQ_10_, MMA, and SDMA after 21 days of CoQ_10_ supplementation were significantly higher than those in the placebo group.

### 3.3. Changes in Vitamin D and Amino Acid Metabolites before the 21-Day Supplementation Period

**Figure 2:**
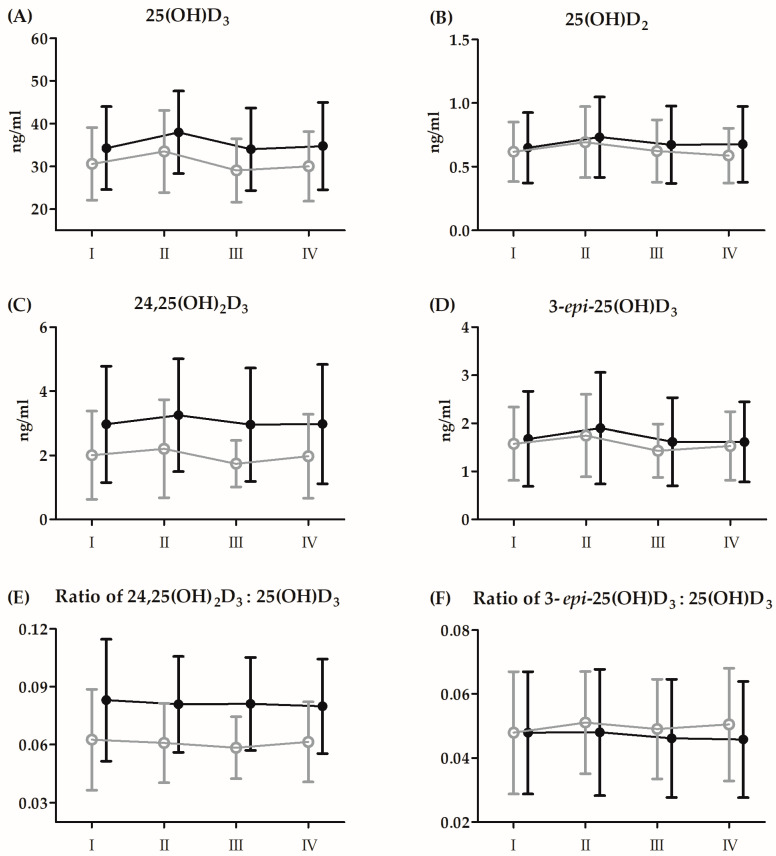
Changes in serum levels of vitamin D metabolites induced by Bruce protocol treadmill test (BTT) before 21 days of supplementation with CoQ_10_ (mean *±* SD). (**A**) 25(OH)D_3_, 25-hydroxyvitamin D_3_; (**B**) 25(OH)D_2_, 25-hydroxyvitamin D_2_; (**C**) 24,25(OH)_2_D_3_, 24,25-dihydroxyvitamin D_3_; (**D**) 3-*epi*-25(OH)D_3_, 3-epi-25-hydroxyvitamin D_3_; (**E**) Ratio 24,25(OH)_2_D_3_:25(OH)D_3_, 24,25-dihydroxyvitamin D_3_:25-hydroxyvitamin D_3_; (**F**) Ratio 3-*epi*-25(OH)D_3_:25(OH)D_3_, 3-epi-25-hydroxyvitamin:25-hydroxyvitamin D_3_. Black circles, supplementation group (n = 14); grey circles, placebo group (n = 14): I, baseline; II, immediately after BTT; III, 3 h after BTT; IV, 24 h after BTT.

The results and the ANOVA outcomes of changes in Vitamin D_3_ induced by BTT before 21 days of CoQ_10_ supplementation are presented in Figure 2 and Table 4, respectively.

Regardless of the group division, significant increases in concentrations of 25(OH)D_3_ (10.8%, *p* < 0.01), 25(OH)D_2_ (12.4%, *p* < 0.01), and 3-*epi*-25(OH)D_3_ (12.3%, *p* < 0.01) were noted immediately after BTT compared to the baseline. Regardless of the time point of serum measurement, in the supplemented group, the mean values of the ratio of 24,25(OH)_2_D_3_:25(OH)D_3_ concentration were significantly higher compared to the placebo group (25.9%, *p* < 0.05).

**Figure 3:**
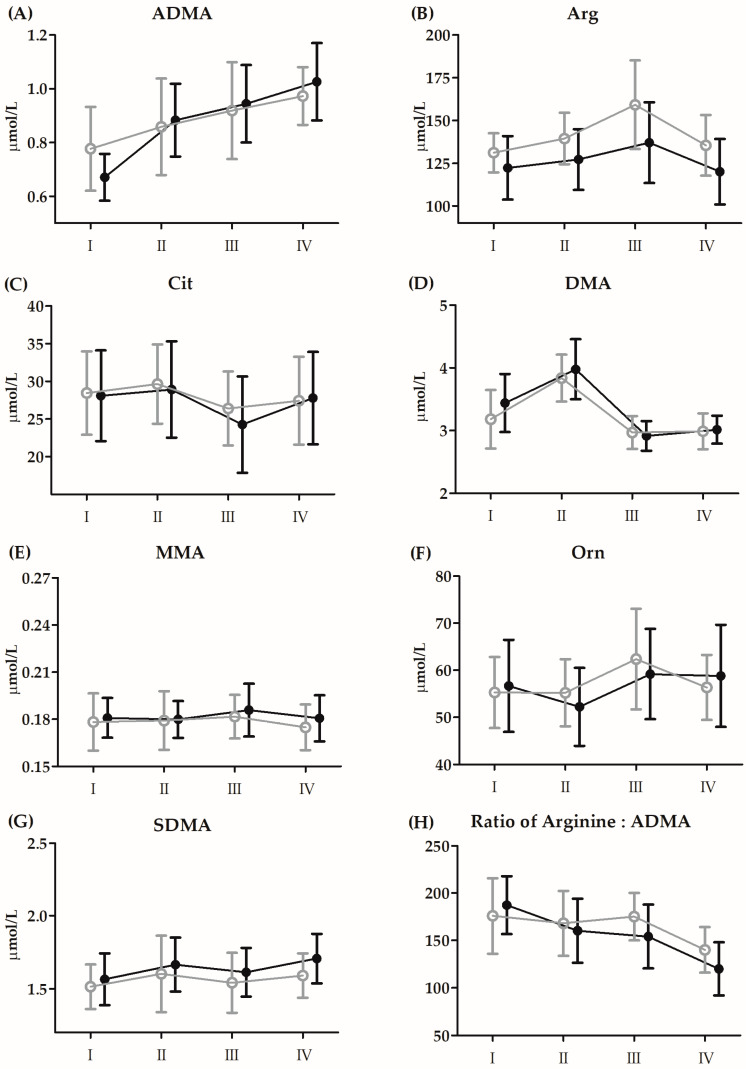
Changes in serum levels of amino acid metabolites induced by Bruce protocol treadmill test (BTT) before 21 days of supplementation with CoQ_10_ (means ± SD). (**A**) asymmetric dimethylarginine (ADMA); (**B**) arginine (Arg); (**C**) citrulline (Cit); (**D**) dimethylamine (DMA); (**E**) methylarginine (MMA); (**F**) ornithine (Orn); (**G**) symmetric dimethylarginine (SDMA); (**H**) ratio of arginine:ADMA. Black circles, supplementation group (n = 14); grey circles, placebo group (n = 14): I, baseline; II, immediately after BTT; III, 3 h after BTT; IV, 24 h after BTT.

The results and ANOVA outcomes of changes in amino acid metabolites induced by BTT before 21 days of CoQ_10_ supplementation are presented in Figure 3 and Table 5, respectively.

Regardless of the group, although there were significant increases in Arg (17.5%, *p* < 0.01) and Orn (7.6%, *p* < 0.05) 3 h after BTT, Cit concentrations significantly decreased (−10.4%, *p* < 0.01) compared to baseline. The concentration of DMA (18.2%, *p* < 0.01) significantly increased immediately after BTT. In turn, the concentrations of ADMA (40.8%, *p* < 0.01) and SDMA (7.2%, *p* < 0.05) increased significantly only after 24 h of exercise.

Regardless of the group allocation, although there were significant increases in Arg (17.5%, *p* < 0.01) and Orn (7.6%, *p* < 0.05) 3 h after BTT, Cit concentrations significantly decreased (−10.4%, *p* < 0.01) compared to baseline. The concentration of DMA (18.2%, *p* < 0.01) significantly increased immediately after BTT. In turn, the concentrations of ADMA (40.8%, *p* < 0.01) and SDMA (7.2%, *p* < 0.05) increased significantly only 24 h after BTT.

### 3.4. Changes in Vitamin D and Amino Acid Metabolites after the 21-Day Supplementation Period

**Figure 4:**
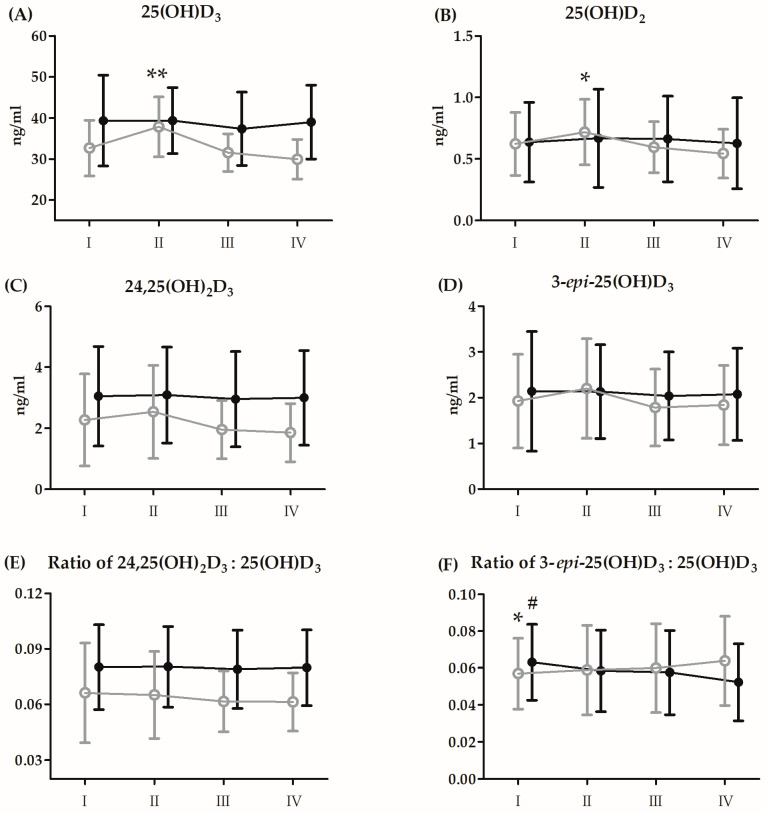
Changes in serum levels of vitamin D metabolites induced by Bruce protocol treadmill test (BTT) after 21 days of supplementation with coenzyme Q_10_ (means and standard deviations). (**A**) 25(OH)D_3_, 25-hydroxyvitamin D_3_; (**B**) 25(OH)D_2_, 25-hydroxyvitamin D_2_; (**C**) 24,25(OH)_2_D_3_, 24,25-dihydroxyvitamin D_3_; (**D**) 3-*epi*-25(OH)D_3_, 3-epi-25-hydroxyvitamin D_3_ (**E**) Ratio 24,25(OH)_2_ D_3_:25(OH)D_3_, 24,25-dihydroxyvitamin D_3_:25-hydroxyvitamin D_3_; (**F**) Ratio 3-*epi*-25(OH)D_3_:25(OH)D_3_, 3-epi-25-hydroxyvitamin D_3_:25-hydroxyvitamin D_3_. Black circles, supplemented group (n = 14); grey circles, placebo (n = 14). I, resting; II, immediately after BTT; III, 3 h post BTT; IV, 24 h post BTT; * significant difference vs. placebo group in IV time point at *p* < 0.05; ** significant difference vs. placebo group in I, III and IV time point at *p* < 0.01; # significant difference vs. CoQ_10_ group in IV time point at *p* < 0.05.

The results and the ANOVA outcomes of changes in Vitamin D_3_ induced by BTT after 21 days of CoQ_10_ supplementation are presented in Figure 4 and Table 6, respectively.

Regardless of the group allocation, significant increases in concentrations of 25(OH)D_3_ (6.8%, *p* < 0.05), 25(OH)D_2_ (10.7%, *p* < 0.05), and 3-*epi*-25(OH)D_3_ (12.3%, *p* < 0.01) were noted immediately after BTT compared to the baseline. Regardless of the time point of serum measurement, in the supplemented group, the mean values of 25(OH)D_3_ (18.3%, *p* < 0.01), 24,25(OH)_2_D_3_ (50.7%, *p* < 0.01) and the ratio of 24,25(OH)_2_D_3_:25(OH)D_3_ (25.5%, *p* < 0.05) concentrations were significantly higher compared to the placebo group.

The significant interaction of group factor and repeated measurement was only observed in 25(OH)D_3_ and 25(OH)D_2_ and is depicted in Figure 4

**Figure 5:**
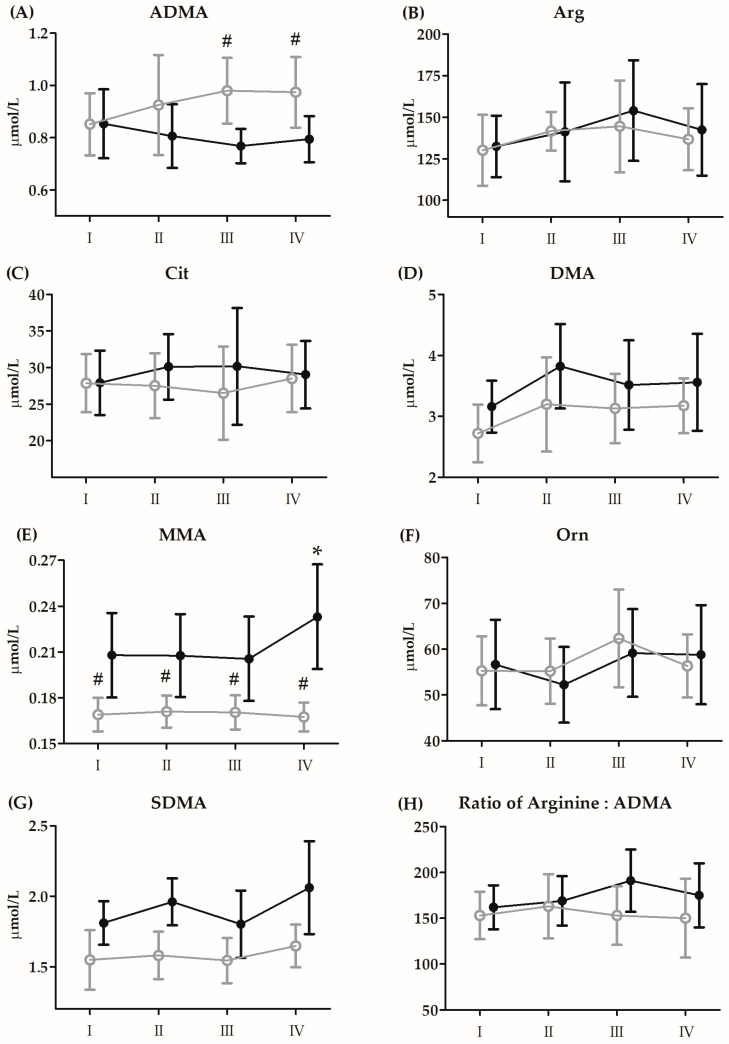
Changes in serum levels of amino acid metabolites induced by Bruce protocol treadmill test (BTT) after 21 days of supplementation with coenzyme Q_10_ (means and standard deviations). (**A**) asymmetric dimethylarginine (ADMA); (**B**) arginine (Arg); (**C**) citrulline (Cit); (**D**) dimethylamine (DMA); (**E**) methylarginine (MMA); (**F**) ornithine (Orn); (**G**) symmetric dimethylarginine (SDMA); (**H**) ratio of arginine:ADMA. Black circles, supplemented group (n = 14); grey circles, placebo (n = 14). I, resting; II, immediately after BTT; III, 3 h post BTT; IV, 24 h post BTT; * significant difference vs. supplemented group in all timepoints at *p* < 0.01; # significant difference vs. supplemented group at *p* < 0.01.

The results and ANOVA outcomes of changes in amino acid metabolites induced by BTT after 21 days of CoQ_10_ supplementation are presented in Figure 5 and Table 7, respectively.

In the case of DMA (18.0%, *p* < 0.05), MMA (28.1%, *p* < 0.01), SDMA (21.1%, *p* < 0.01) and Arg:ADMA ratio (12.5%, *p* < 0.05), the concentration values recorded in the supplemented group, regardless of the time point of serum collected, turned out to be significantly higher compared to the placebo group. In turn, the ADMA (13.8%, *p* < 0.01) concentration values in the supplemented group were significantly lower compared to the placebo group.

A significant interaction of both factors was noted only in ADMA (*p* < 0.01, η^2^ = 0.15) and MMA and is depicted in Figure 5.

## 4. Discussion

The present study revealed that CoQ_10_ supplementation significantly influenced exercise-induced changes in vitamin D and methylated derivatives of arginine metabolism in young men. Interestingly, baseline serum concentrations of ADMA and SDMA, but not MMA, increased after exercise. ADMA and MMA were mainly eliminated by intracellular conversion to Cit and DMA in a reaction catalysed by DDAH since around 20% of ADMA is excreted by the kidneys [7].

Conversely, most SDMA is removed from the body by renal excretion. Thus, the serum concentration of MMA is a net result of its formation and elimination, and the formation of methylated arginine derivatives is related to protein proteolysis. Protein-containing arginine residues can undergo a methylation reaction catalysed by a family of enzymes known as protein arginine methyltransferases [14], which can be released during proteolysis. Some forms of exercise can increase skeletal muscle proteolysis; for example, the expression of two muscle-specific ubiquitin ligase genes—MuRF1 and atrogin-1—increases immediately after a run, suggesting increased postexercise proteasome-mediated proteolysis [15]. In this study, the ADMA concentration increased in 3 h and 24 h after exercise, perhaps due to this process. Conversely, likely, the elimination of ADMA and MMA by DDAH and SDMA in the kidneys can also be modulated by exercise. An increase in DMA immediately after exercise indicated that the reaction catalysed by DDAH was augmented. Conversely, the accumulation of SDMA indicated that renal excretion was insufficient to stabilise the serum concentration. As mentioned previously, DDAH enzymatic activity can be inhibited by the increased formation of ROS [7], which can occur during exercise [5], potentially influencing DDAH activity. A study on an animal model demonstrated that increasing the GSH concentration in diabetic rats restored the DDAH protein level [16]. Our results partially support this observation since, in the CoQ_10_ supplementation group, the ADMA level decreased, but it increased in the placebo group. Conversely, MMA increased 24 h after exercise in the CoQ_10_ group but not in the placebo group. Considering that resting serum concentrations of SDMA and MMA increased significantly after supplementation with CoQ_10_, it can be assumed that some changes in renal excretion were responsible for the observed differences. ADMA directly and SDMA as a competitive inhibitor of L-arginine transport [17] inhibit reactions catalysed by nitric oxide synthase (NOS). Since ADMA is a competitive inhibitor of NOS, the arginine/ADMA ratio is an essential determinant of NO formation. It is important to note that the Arg:ADMA ratio was higher 3 h after exercise in the CoQ_10_ group than in the placebo group. It may be that the antioxidant properties of CoQ_10_ were responsible for the observed effects. In summary, our results indicate that CoQ_10_ can modify the serum concentrations of MMAs. However, it should be noted that all concentrations of MMA were within the range of values observed in healthy individuals [18,19].

Studies have demonstrated interconnections between vitamin D and oxidative stress. Vitamin D can exert some antioxidative effects; for example, lower oxidative damage to proteins and lipids has been observed in the skeletal muscle of vitamin D-supplemented patients [20]. Vitamin D can reduce oxidative stress by influencing antioxidant enzyme activity or reducing inflammation. Conversely, oxidative stress can affect the vitamin D activation process and biological action. For example, a stress condition accompanied by a low GSH level impairs the activity of 25 hydroxylase [21]. Moreover, oxidative stress can lead to the downregulation of vitamin D receptor (VDR) and 1alpha hydroxylase but be reversed by cysteine, which is essential for GSH synthesis. In our study, we speculated that CoQ_10_ would influence the vitamin D metabolite changes induced by exercise. This demonstrated that CoQ_10_ supplementation abrogated increased serum 25(OH)D_3_, 24,25(OH)_2_D_3_, and 3-*epi*-25(OH)D_3_ concentrations after exercise, even if such an increase was observed at baseline and in the placebo group.

## 5. Conclusions

The present study revealed that CoQ_10_ supplementation significantly influenced exercise-induced changes in vitamin D and methylated derivatives of arginine metabolism in young men. Moreover, CoQ_10_ was shown to increase the resting level of serum arginine. Increased serum vitamin D after exercise has been reported in many studies [22,23]. However, this is the first report demonstrating that this process is inhibited by the antioxidant CoQ_10_. These data indicate that CoQ_10_ significantly modified the exercise-induced changes in vitamin D metabolism; however, the physiological meaning of this needs further studies. Moreover, CoQ_10_ supplementation increases the Arg to ADMA ratio, suggesting that in the supplemented athletes, the formation of nitric oxide will be facilitated, which can positively influence exercise performance.

## 6. Limitations

The current study has some potential limitations, the first of which is the relatively small sample size. Further studies should employ increased sample sizes and/or crossover study designs. Second, the study did not include any control of the dietary regime, and potential differences in the characteristics of meals rich in CoQ_10_ (e.g., fish such as sardines, mackerel, salmon, natural oils, spinach, whole grain products, and broccoli) might have interfered with the applied intervention. However, the observed change in the CoQ_10_ serum concentration (a 2.76 fold serum increase) was directly caused by the supplementation.

In addition, future researchers should use muscle biopsies to assess changes in CoQ_10_ concentrations in muscle tissue. This would allow for more detailed conclusions regarding the observed reactions.

## Figures and Tables

**Table 1:** Bruce Treadmill Test Stages, Speeds, and Inclines.

Stage	Treadmill Speed	Treadmill Incline
**1**	2.74 km/h (1.7 mph)	10% grade
**2**	4.03 km/h (2.5 mph)	12% grade
**3**	5.47 km/h (3.4 mph)	14% grade
**4**	6.76 km/h (4.2 mph)	16% grade
**5**	8.05 km/h (5.0 mph)	18% grade
**6**	8.86 km/h (5.5 mph)	20% grade
**7**	9.66 km/h (6.0 mph)	22% grade
**8**	10.46 km/h (6.5 mph)	24% grade
**9**	11.26 km/h (7.0 mph)	26% grade
**10**	12.07 km/h (7.5 mph)	28% grade

**Note:** Mph, miles per hour.

**Table 2:** Physical and physiological characteristics of the participants before and after 21 days of supplementation with CoQ_10_.

Variable	Unit	Supplementation Group (n = 14)		Placebo Group (n = 14)	
		Before	After	Before	After
		Mean ± SD	Mean ± SD	Mean ± SD	Mean ± SD
Height	cm	179.1 ± 7.3	-	181.2 ± 8.1	-
Body mass	kg	77.7 ± 7.1	76.0 ± 6.8	79.9 ± 7.8	79.4 ± 7.3
Body fat percentage	%	12.0 ± 4.6	11.5 ± 4.3	12.8 ± 5.1	12.4 ± 5.0
Body mass index	kg/m^2^	23.5 ± 3.2	23.1 ± 3.0	24.0 ± 3.6	23.7 ± 3.6
Maximal oxygen uptake	mL/min/kg	57.2 ± 8.0	55.9 ± 7.9	51.3 ± 9.4	52.8 ± 7.5
Respiratory exchange ratio		1.26 ± 0.05	1.28 ± 0.07	1.29 ± 0.07	1.28 ± 0.06
Maximal heart rate	beats/min	197.4 ± 7.5	197.7 ± 6.3	193.9 ± 5.2	192 ± 6.7

**Table 3:** Baseline concentrations of CoQ_10_, vitamin D_3_ metabolites, and amino acid metabolites before and after 21-day supplementation with CoQ_10_.

Variable	Unit	Supplementation Group (n = 14)		Placebo Group (n = 14)	
		Before	After	Before	After
		Mean ± SD	Mean ± SD	Mean ± SD	Mean ± SD
CoQ_10_	ng/mL	900 ± 321	2486 ± 1180 *†	849 ± 187	827 ± 166
25(OH)D_3_	ng/mL	34.0 ± 9.0	39.5 ± 10.8 *	30.5 ± 8.0	32.3 ± 7.0
25(OH)D_2_	ng/mL	0.6 ± 0.3	0.7 ± 0.3	0.6 ± 0.2	0.6 ± 0.3
24,25(OH)_2_D_3_	ng/mL	3.0 ± 1.8	3.3 ± 2.3	2.1 ± 1.4	2.3 ± 1.5
3-*epi*-25(OH)D_3_	ng/mL	1.7 ± 1.0	2.6 ± 1.4 *	1.6 ± 0.8	1.9 ± 0.8
Ratio 24,25(OH)_2_D_3_: 25(OH)D_3_		0.08 ± 0.03	0.08 ± 0.02	0.06 ± 0.02	0.06 ± 0.03
Ratio 3-*epi*-25(OH)D_3_: 25(OH)D_3_		0.04 ± 0.02	0.06 ± 0.02 *	0.05 ± 0.02	0.06 ± 0.02
ADMA	µmol/L	0.65 ± 0.08	0.85 ± 0.13	0.77 ± 0.15	0.85 ± 0.11
Arg	µmol/L	122.3 ± 18.5	136.9 ± 19.8 *	131.2 ± 11.5	129.2 ± 20.9
Cit	µmol/L	28.4 ± 5.5	29.9 ± 4.2	28.4 ± 5.5	27.6 ± 3.9
DMA	µmol/L	3.4 ± 0.5	3.2 ± 0.4	3.2 ± 0.5	2.7 ± 0.5
MMA	µmol/L	0.17 ± 0.01	0.21 ± 0.03 *†	0.17 ± 0.02	0.17 ± 0.02
Orn	µmol/L	60.0 ± 11.0	63.6 ± 11.1	55.3 ± 4.6	56.7 ± 8.5
SDMA	µmol/L	1.6 ± 0.2	1.8 ± 0.2 *†	1.5 ± 0.2	1.5 ± 0.3
Ratio Arg:ADMA		183 ± 30.7	162.6 ± 24.8	175.9 ± 40.0	156.3 ± 26.8

Note: markers—CoQ_10_, Coenzyme Q_10_; 25(OH)D_3_, 25-hydroxyvitamin D_3_; 25(OH)D_2_, 25-hydroxyvitamin D_2_; 24,25(OH)_2_D_3_, 24,25-dihydroxyvitamin D_3_; 3-*epi*-25(OH)D_3_, 3-epi-25-hydroxyvitamin D_3_; ADMA, asymmetric dimethylarginine; Arg, arginine; Cit, citrulline; DMA, dimethylamine; MMA, methylarginine; Orn, ornithine; SDMA, symmetric dimethylarginine. * significant difference versus the supplementation group before 21-day supplementation with CoQ_10_; † significant difference versus the placebo group after 21-day supplementation with CoQ_10_.

**Table 4:** Two-way ANOVA (2 groups × 4 repeated measures) of changes in serum levels of vitamin D metabolites induced by Bruce protocol treadmill test (BTT) before 21 days of supplementation with CoQ_10_.

Variable	Effect	F	Df	P	Effect Size (η^2^)	Post-Hoc Outcome
25(OH)D_3_	GR	1.87	1, 26	0.18	0.06	II > I, III, IV
	RM	11.78	3, 78	<0.01 **	0.31	
	GR × RM	0.27	3, 78	0.84	0.01	
25(OH)D_2_	GR	0.26	1, 26	0.61	0.01	II > I, III, IV
	RM	14.57	3, 78	<0.01 **	0.35	
	GR × RM	1.63	3, 78	0.18	0.05	
24,25(OH)_2_D_3_	GR	3.32	1, 26	0.08	0.11	II > III
	RM	3.49	3, 78	0.02 *	0.12	
	GR × RM	0.43	3, 78	0.73	0.01	
3-*epi*-25(OH)D_3_	GR	0.16	1, 26	0.68	0.01	II > I, III, IV
	RM	9.27	3, 78	<0.01 **	0.26	
	GR × RM	0.29	3, 78	0.83	0.01	
24,25(OH)_2_D_3_:25(OH)D_3_	GR	5.56	1, 26	0.03 *	0.17	S > C
	RM	0.86	3, 78	0.46	0.03	
	GR × RM	0.41	3, 78	0.74	0.02	
3-*epi*-25(OH)D_3_:25(OH)D_3_	GR	0.29	1, 26	0.59	0.01	I, II, IV > III
	RM	4.31	3, 78	<0.01 **	0.14	
	GR × RM	0.62	3, 78	0.60	0.02	

Note: Study design: 25(OH)D_3_, 25-hydroxyvitamin D_3_; 25(OH)D_3_, 25-hydroxyvitamin D_2_; 24,25(OH)_2_D_3_, 24,25-dihydroxyvitamin D_3_; 3-*epi*-25(OH)D_3_, 3-epi-25-hydroxyvitamin D_3_; GR, group; RM, repeated measures; Effect Size (η^2^), Partial Eta Squared; Df, degrees of freedom; F-value is the ratio of two variances. I, before BTT; II, immediately after the BTT; III, 3 h after the BTT; and IV, 24 h after BTT. Significant difference detected at * *p* ≤ 0.05 or ** *p* ≤ 0.01.

**Table 5:** Two-way ANOVA (2 groups × 4 repeated measures) of changes in serum levels of amino acid metabolites induced by Bruce protocol treadmill test (BTT) before 21 days of supplementation with CoQ_10_.

Variable	Effect	F	Df	P	Effect Size (η^2^)	Post-Hoc Outcome
ADMA	GR	0.01	1, 26	0.98	<0.01	I < II, III, IV
	RM	25.88	3, 78	<0.01 **	0.43	
	GR × RM	0.24	3, 78	0.07	0.06	
Arg	GR	3.49	1, 26	0.07	0.11	III > I, II, IV
	RM	15.60	3, 78	<0.01 **	0.37	
	GR × RM	0.63	3, 78	0.59	0.02	
Cit	GR	0.12	1, 26	0.72	<0.01	III > I, II, IV
	RM	10.81	3, 78	<0.01 **	0.29	
	GR × RM	1.05	3, 78	0.37	0.04	
DMA	GR	0.24	1, 26	0.42	0.02	II > I > II, IV
	RM	99.03	3, 78	<0.01 **	0.79	
	GR × RM	2.36	3, 78	0.07	0.08	
MMA	GR	0.49	1, 26	0.48	0.01	
	RM	1.71	3, 78	0.17	0.06	
	GR × RM	0.30	3, 78	0.82	0.01	
Orn	GR	0.06	1, 26	0.80	<0.01	III > I, II
	RM	6.14	3, 78	<0.01 **	0.15	
	GR × RM	1.50	3, 78	0.21	0.04	
SDMA	GR	2.61	1, 26	0.11	0.09	I < IV
	RM	2.69	3, 78	0.05 *	0.09	
	GR × RM	0.21	3, 78	0.88	0.01	
Arg:ADMA	GR	1.23	1, 26	0.27	0.05	I, II, III > IV
	RM	19.64	3, 78	<0.01 **	0.43	
	GR × RM	2.53	3, 78	0.07	0.08	

Note: Study design: GR, group; RM, repeated measures; Effect Size (η^2^); Partial Eta Squared; Df, degrees of freedom; F-value is the ratio of two variances; ADMA, asymmetric dimethylarginine; Arg, arginine; Cit, citrulline; DMA, dimethylamine; MMA, methylarginine; Orn, ornithine; SDMA, symmetric dimethylarginine; I, before the BTT; II, immediately after the BTT; III, 3 h after the BTT and IV, 24 h after BTT. Significant difference detected at * *p* ≤ 0.05 or ** *p* ≤ 0.01.

**Table 6:** Two-way ANOVA (2 groups × 4 repeated measures) of changes in serum levels of vitamin D metabolites induced by Bruce protocol treadmill test (BTT) after 21 days of supplementation with CoQ_10_.

Variable	Effect	F	Df	P	Effect Size (η^2^)	Post-Hoc Outcome
25(OH)D_3_	GR	4.56	1, 26	0.04 *	0.14	S > P
	RM	8.67	3, 78	<0.01 **	0.25	II > I, III
	GR × RM	5.25	3, 78	<0.01 **	0.16	PII > PI, PIII, PIV
25(OH)D_2_	GR	0.75	1, 26	0.39	0.03	
	RM	5.03	3, 78	<0.01 **	0.16	II > I, III, IV
	GR × RM	3.00	3, 78	0.03 *	0.11	PII > PIV
24,25(OH)_2_D_3_	GR	4.96	1, 26	0.03 *	0.16	S > P
	RM	2.97	3, 78	0.04 *	0.11	II > III
	GR × RM	1.28	3, 78	0.28	0.04	
3-*epi*-25(OH)D_3_	GR	0.69	1, 26	0.41	0.02	
	RM	4.13	3, 78	<0.01 **	0.13	II > III, IV
	GR × RM	1.82	3, 78	0.15	0.06	
24,25(OH)_2_D_3_:25(OH)D_3_	GR	5.05	1, 26	0.03 *	0.16	S > P
	RM	0.72	3, 78	0.53	0.02	
	GR × RM	0.34	3, 78	0.79	0.01	
3-*epi*-25(OH)D_3_:25(OH)D_3_	GR	0.01	1, 26	0.97	<0.01	
	RM	0.46	3, 78	0.70	0.02	
	GR × RM	3.90	3, 78	0.01 *	0.12	SI > SIV; PI < PIV

Note: Study design: 25(OH)D_3_, 25-hydroxyvitamin D_3_; 25(OH)D_2_, 25-hydroxyvitamin D_2_; 24,25(OH)_2_D_3,_ 24,25-dihydroxyvitamin D_3_; 3-*epi*-25(OH)D_3_, 3-epi-25-hydroxyvitamin D_3_; GR, group; RM, repeated measures; Effect Size (η^2^); Partial Eta Squared; Df, degrees of freedom; F-value is the ratio of two variances; S, supplementation group (n = 14); P, placebo group (n = 14); I, before BTT; II, immediately after the BTT; III, 3 h after the BTT and IV, 24 h after BTT. Significant difference detected at * *p* ≤ 0.05 or ** *p* ≤ 0.01.

**Table 7:** Two-way ANOVA (2 groups × 4 repeated measures) of changes in serum levels of amino acid metabolites induced by Bruce protocol treadmill test (BTT) after the 21 days of supplementation with CoQ_10_.

Variable	Effect	F	Df	P	Effect Size (η^2^)	Post-Hoc Outcome
ADMA	GR	17.59	1, 26	<0.01 **	0.4	S < P
	RM	0.39	3, 78	75	0.01	
	GR × RM	4.82	3, 78	<0.01 **	0.15	SIII, SIV < PIII, PIV
Arg	GR	0.31	1, 26	0.58	0.01	I < III
	RM	3.51	3, 78	0.02 *	0.11	
	GR × RM	1.63	3, 78	0.18	0.05	
Cit	GR	2.79	1, 26	0.10	0.09	
	RM	2.08	3, 78	0.10	0.07	
	GR × RM	0.11	3, 78	0.95	<0.01	
DMA	GR	4.78	1, 26	0.42	0.15	S > P
	RM	8.66	3, 78	<0.01 **	0.24	I < II, III, IV
	GR × RM	0.46	3, 78	0.07	0.01	
MMA	GR	24.18	1, 26	<0.01 **	0.48	S > P
	RM	15.39	3, 78	<0.01 **	0.37	I, II, III < IV
	GR × RM	24.51	3, 78	<0.01 **	0.48	SI–SIV > PI–PIV
Orn	GR	0.82	1, 26	0.37	0.03	II < III
	RM	3.84	3, 78	<0.01 **	0.13	
	GR × RM	0.83	3, 78	0.47	0.03	
SDMA	GR	29.54	1, 26	<0.01 **	0.53	S > P
	RM	6.58	3, 78	<0.01 **	0.2	I < IV
	GR × RM	1.45	3, 78	0.23	0.05	
Arg/ADMA	GR	5.63	1, 26	0.03 *	0.18	S > P
	RM	1.09	3, 78	0.35	0.04	
	GR × RM	1.54	3, 78	0.20	0.06	

Note: Study design: GR, group; RM, Repeated measure; Effect Size (η^2^); Partial Eta Squared; Df degrees of freedom; F-value is the ratio of two variances; S, supplementation group (n = 14); P, placebo group (n = 14); ADMA, asymmetric dimethylarginine; Arg, arginine; Cit, citrulline; DMA, dimethylamine; MMA, methylarginine; Orn, ornithine; SDMA, symmetric dimethylarginine; I, before BTT; II, immediately after the BTT; III, 3 h after the BTT and IV, 24 h after BTT. Significant difference detected at * *p* ≤ 0.05 or ** *p* ≤ 0.01.

## Data Availability

All the experimental data are available from the corresponding authors.
